# Multiparametric MR and PET Imaging of Intratumoral Biological Heterogeneity in Patients with Metastatic Lung Cancer Using Voxel-by-Voxel Analysis

**DOI:** 10.1371/journal.pone.0132386

**Published:** 2015-07-17

**Authors:** Stephan Metz, Carl Ganter, Sylvie Lorenzen, Sandra van Marwick, Konstantin Holzapfel, Ken Herrmann, Ernst J. Rummeny, Hans-Jürgen Wester, Markus Schwaiger, Stephan G. Nekolla, Ambros J. Beer

**Affiliations:** 1 Department of Radiology of the Technische Universitaet Muenchen, Munich, Germany; 2 Department of Hemato-Oncology of the Technische Universitaet Muenchen, Munich, Germany; 3 Departments of Nuclear Medicine of the Technische Universitaet Muenchen, Munich, Germany; 4 Institute for Radiopharmaceutical Chemistry, Technische Universitaet Muenchen, Garching, Germany; Universidad Carlos III of Madrid, SPAIN

## Abstract

**Objectives:**

Diffusion-weighted magnetic resonance imaging (DW-MRI) and imaging of glucose metabolism by positron emission tomography (FDG-PET) provide quantitative information on tissue characteristics. Combining the two methods might provide novel insights into tumor heterogeneity and biology. Here, we present a solution to analyze and visualize the relationship between the apparent diffusion coefficient (ADC) and glucose metabolism on a spatially resolved voxel-by-voxel basis using dedicated quantitative software.

**Materials and Methods:**

In 12 patients with non small cell lung cancer (NSCLC), the primary tumor or metastases were examined with DW-MRI and PET using ^18^F-fluorodeoxyglucose (FDG). The ADC’s from DW-MRI were correlated with standardized-uptake-values on a voxel-by-voxel basis using custom made software (Anima M^3^P). For cluster analysis, we used prospectively defined thresholds for ^18^F-FDG and ADC to define tumor areas of different biological activity.

**Results:**

Combined analysis and visualization of ADC maps and PET data was feasible in all patients. Spatial analysis showed relatively homogeneous ADC values over the entire tumor area, whereas FDG showed a decreasing uptake towards the tumor center. As expected, restricted water diffusivity was notable in areas with high glucose metabolism but was also found in areas with lower glucose metabolism. In detail, 72% of all voxels showed low ADC values (<1.5x10^-3^ mm^2^/s) and high tracer uptake of ^18^F-FDG (SUV>3.6). However, 83% of the voxels with low FDG uptake also showed low ADC values, increasingly towards the tumor center.

**Conclusions:**

Multiparametric analysis and visualization of DW-MRI and FDG-PET is feasible on a spatially resolved voxel-by-voxel respectively cluster basis using dedicated imaging software. Our preliminary data suggest that water diffusivity and glucose metabolism in metastatic NSCLC are not necessarily correlated in all tumor areas.

## Introduction

Due to the advances in imaging technology, there is an increasing opportunity to perform multiparametric oncological imaging resulting in multiple quantitative parameters that reflect different aspects of tumor biology. This multiparametric approach allows for noninvasive phenotyping of tumor biology which, by combining different functional and molecular imaging methods, might lead to a higher accuracy for tumor detection, prognostic stratification, biopsy and therapy planning, as well as response prediction and early response evaluation in cancer patients [1] [2]. In this context, molecular imaging of certain biomarkers of tumor biology has several advantages compared to histopathological analysis and thus might be an interesting adjunct to histopathology because imaging provides in vivo information without tissue damage, in not accessible tumor parts, and also allows for assessment of temporal changes of quantitative biomarkers by serial imaging. Moreover, it allows for visualization of the biological heterogeneity of tumors.

One promising method for imaging of tumor biology is diffusion-weighted magnetic resonance imaging (DW-MRI), which is increasingly used for lesion detection, staging and response evaluation in cancer patients [3] [4]. As a PET biomarker of biological tumor activity, the radiotracer ^18^F-fluorodeoxyglucose (FDG) is widely used for tumor staging and response assessment [5]. ^18^F-FDG assesses the tumor glucose metabolism and is correlated with vital tumor tissue and aggressiveness [6]. Combined use of DW-MRI and FDG-PET within one imaging session might become increasingly used with the recent introduction of hybrid PET/MR scanners [7] [8]. Although a certain overlap of the information of ^18^F-FDG PET and DW-MRI may be hypothesized, both parameters are based on completely different biophysiological processes, thus their combination might provide complementary information on tumor biology and heterogeneity [2].

In this technical report, we present a software solution for a voxel-by-voxel correlation of DW-MRI and FDG-PET data to analyze the spatial distribution and correlation of parameters derived by both imaging modalities. Moreover, it also allows for visualization of the combined data and overlay over the anatomical data. The perspective is to use this methodology in hybrid PET/MR scanners for further detailed evaluation of this novel combined parameter of tumor biology, e.g. for assessment of patient prognosis, treatment planning or response evaluation.

Thus, in this study, we present a solution to analyze and visualize the relationship between the apparent diffusion coefficient (ADC) and glucose metabolism on a spatially resolved voxel-by-voxel basis using dedicated quantitative software.

## Materials and Methods

### Patients

The study was approved by the ethics committee of the Technische Universitaet Muenchen. Informed written consent was obtained from all patients. 12 chemo-naïve patients with histologically proven metastatic NSCLC were included in the study (4 female, 8 male; mean age 65±2 years; range 52–80 years). This is a subpopulation from a patient collective, which was also part of a former study [9] [10]. Further inclusion criteria were age over 18 years and the ability to give written and informed consent. Exclusion criteria were pregnancy, lactation period, and impaired renal function (serum creatinine level > 1.2 mg/dl). The mean time interval between both examinations was 2.2 days.

### DW-MRI

All MR measurements were performed on a clinical 1.5 T scanner (Magnetom Avanto; Siemens Medical Solutions, Erlangen, Germany) with phased-array body coils. DW images were acquired using a single-shot echo-planar imaging sequence (SSEPI). The diffusion weighting factors (*b* values) were 50, 300, and 600 s/mm^2^. The technical parameters were as follows: echo time 76 ms; echo train length 175; echo spacing 0.83 ms; spectral fat saturation; field of view 262×350 mm; matrix 108×192; NSA 1; section thickness 5 mm; no gap. For shortening of the echo train length, integrated parallel imaging techniques (iPAT) by means of generalized autocalibrating partially parallel acquisitions (GRAPPA) with a 2-fold acceleration factor were used. For respiratory triggering, prospective acquisition correction (PACE) was implemented. Data was acquired during the end-expiratory phase. Apparent diffusion coefficient (ADC) maps were automatically reconstructed for all diffusion-weighted images. ADC values were calculated using signal intensities on the *b* = 50 s/mm^2^ and the *b* = 600 s/mm^2^ images according to the formula:

$$ADC=\left(In\;SI_{50}/SI_{600}\right)\;/\;\left(600-50\right)\;\left(mm^{2}/s\right)$$

where SI_50_ and SI_600_ are signal intensities on the *b* = 50 s/mm^2^ and the *b* = 600 s/mm^2^ images, respectively.

### Radiopharmaceuticals

Synthesis of the precursor and subsequent ^18^F-labeling of FDG was carried out as described previously [11] [12].

### ^18^F-FDG PET Imaging

Uptake of the ^18^F-FDG was determined with a Biograph Sensation 16 PET/CT scanner which incorporates an ACCEL PET system (CTI / Siemens) and a 16-slice multidetector CT (Siemens, Forchheim, Germany). The radiotracer (456±25 MBq) was injected to the patients after 6 hrs of fasting. None of the patients were diabetic or had a fasting blood glucose level above 120 mg/dl. Data acquisition was started 64±3 min after ^18^F-FDG administration. An emission scan was performed from the head to the pelvis (three dimensional mode; 7–8 bed positions, 2 minutes per bed position). Subsequently, an unenhanced low-dose CT scan (120 kV, 26 mAs, collimation 16x0.75 mm) was carried-out in shallow expiration. For attenuation correction, the CT data were converted from Hounsfield units (HU) to linear attenuation coefficients for 511 keV using a single CT energy scaling method based on a bilinear transformation. Emission data were corrected for randoms, dead time, scatter and attenuation and the same reconstruction algorithm was applied as for the conventional PET data.

### Image Analysis

**Fig 1:**
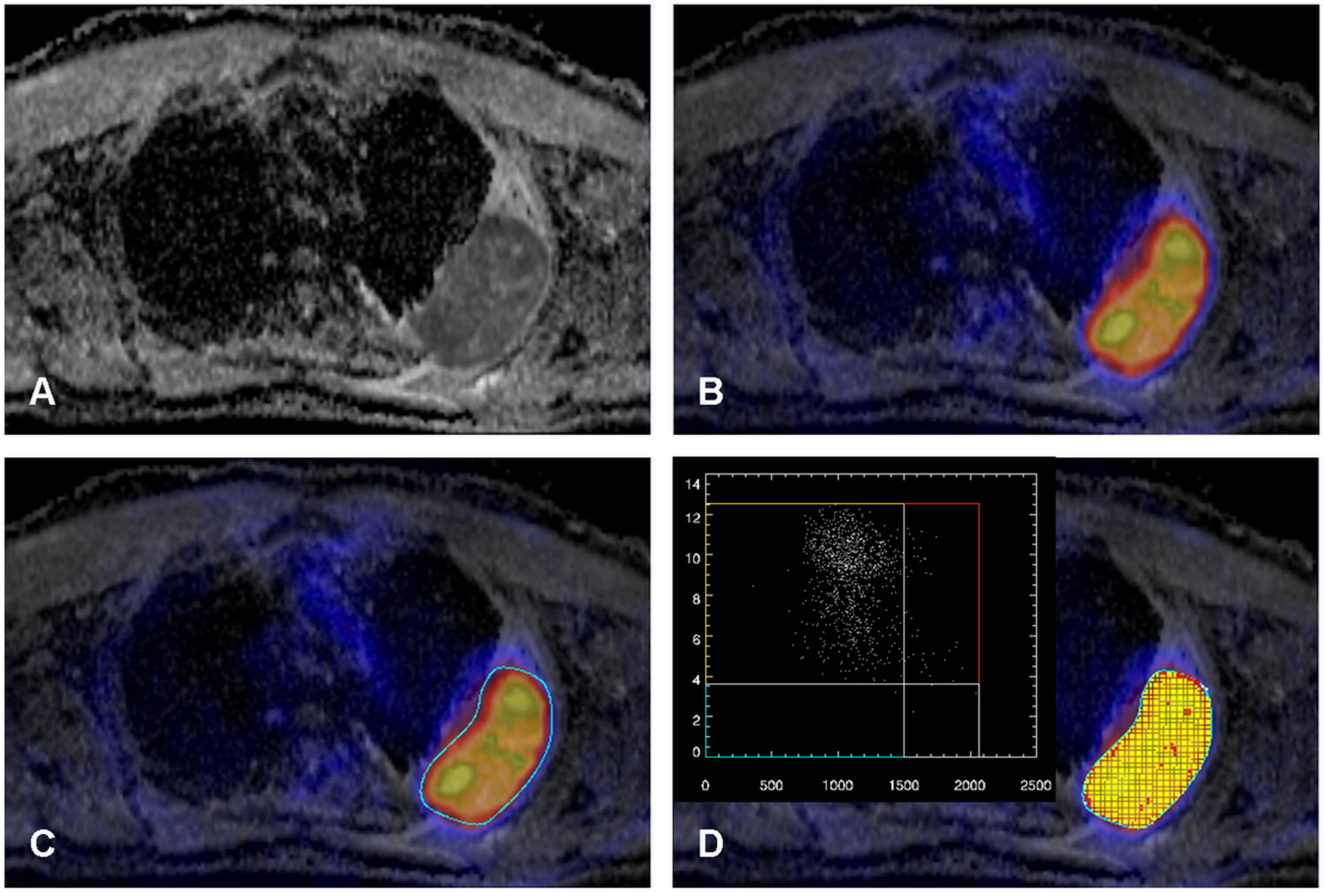
A-D. Patient with a primary NSCLC of the left upper lobe infiltrating the chest wall (A: ADC map from DW-MRI). The tumor shows relatively homogenously low ADC values and high ^18^F-FDG uptake (B image fusion of ^18^F-FDG PET and the ADC map) with some focal spots. A ROI is placed around the tumor (C) and the Anima M^3^P software displays a scatter plot of the voxels within the ROI (D). Thresholds for low and high tracer uptake (y-axis) and low and high ADC values (x-axis) were defined (SUV = 3.6 for ^18^F-FDG and ADC 1.5 x10^-3^ mm²/s) and the localization of the voxels of each quadrant (yellow: SUV_high_/ADC_low_; red: SUV_high_/ADC_high_; blue: SUV_low_/ADC_low_; gray: SUV_low_/ADC_high_) can be displayed in the fusion image (D). Note, most tumor parts show intense glucose metabolism with restricted water diffusivity (yellow), however some spots of the tumor also show intense glucose metabolism despite less restricted water diffusivity (red).

The corrected emissions scans were calibrated to standardized uptake values (SUV; measured activity concentration (Bq/ml) x body weight (g) / injected activity (Bq)) [13]. Data analysis was performed using custom software developed at our institution in order to efficiently manage arbitrary, tomographic multifunctional data sets (Anima M^3^P) [14] [10]. The used software handles all its data in their real, actual 3 dimensional coordinate space (metric units), thus keeping the true dimensions intact, even with arbitrary multifunctional data. Image fusion of the parametric ADC maps to the ^18^F-FDG PET data sets was performed in the same window, upscaling the PET data sets to the resolution of the ADC maps by linear interpolation. This does, of course, imply certain inexactness in the data analysis but it ensures the best possible calculation of MRI based statistical values. The image fusion was done manually and internally adapted with a mathematical trilinear interpolation. Reference anatomical landmarks were manually matched and fused. Subsequently, a region of interest (ROI) was drawn manually covering the entire tumor area on multiple sections. The software then provides the distribution of the quantitative parameters for each imaging method on a voxel-by-voxel basis in the tumor ROI, which can be displayed as correlation of the ADC’s from DW-MRI and the SUV’s from ^18^F-FDG PET in a scatter-plot (Fig 1). The voxel based parametric data were computed and saved for comparative analysis.

### Cluster Analysis

For prospective definition of the threshold for low and high tracer uptake, we used data from previous studies [9]. We decided to use the lower 25^th^ percentile of the previously reported data on tracer distribution of ^18^F-FDG as a threshold for definition of low tracer uptake. This resulted in a threshold at SUV 3.6 for ^18^F-FDG. For ADC, mean data reported in the literature for lung cancer are 1.22±0.19 x 10^−3^ mm^2^/s chemo-naïve and 1.76±0.47 x 10^−3^ mm^2^/s after chemotherapy [15]. Thus we decided to use 1.5x10^-3^ mm^2^/s as threshold, with higher values defining (micro-)necrotic tissue. The definition of the thresholds however is somewhat arbitrary and can be altered by the user according to the focus and the aim of the analysis. By this approach, the total tumor area was divided in up to four tumor regions with different “biological activity”: areas with high ^18^F-FDG uptake and high ADC values (SUV_high_/ADC_high_), areas with either high ^18^F-FDG uptake and low ADC values (SUV_high_/ADC_low_) or vice versa (SUV_low_/ADC_high_), and finally areas with low ^18^F-FDG uptake and low ADC values (SUV_low_/ADC_low_).

### Voxel ring analysis

**Fig 2:**
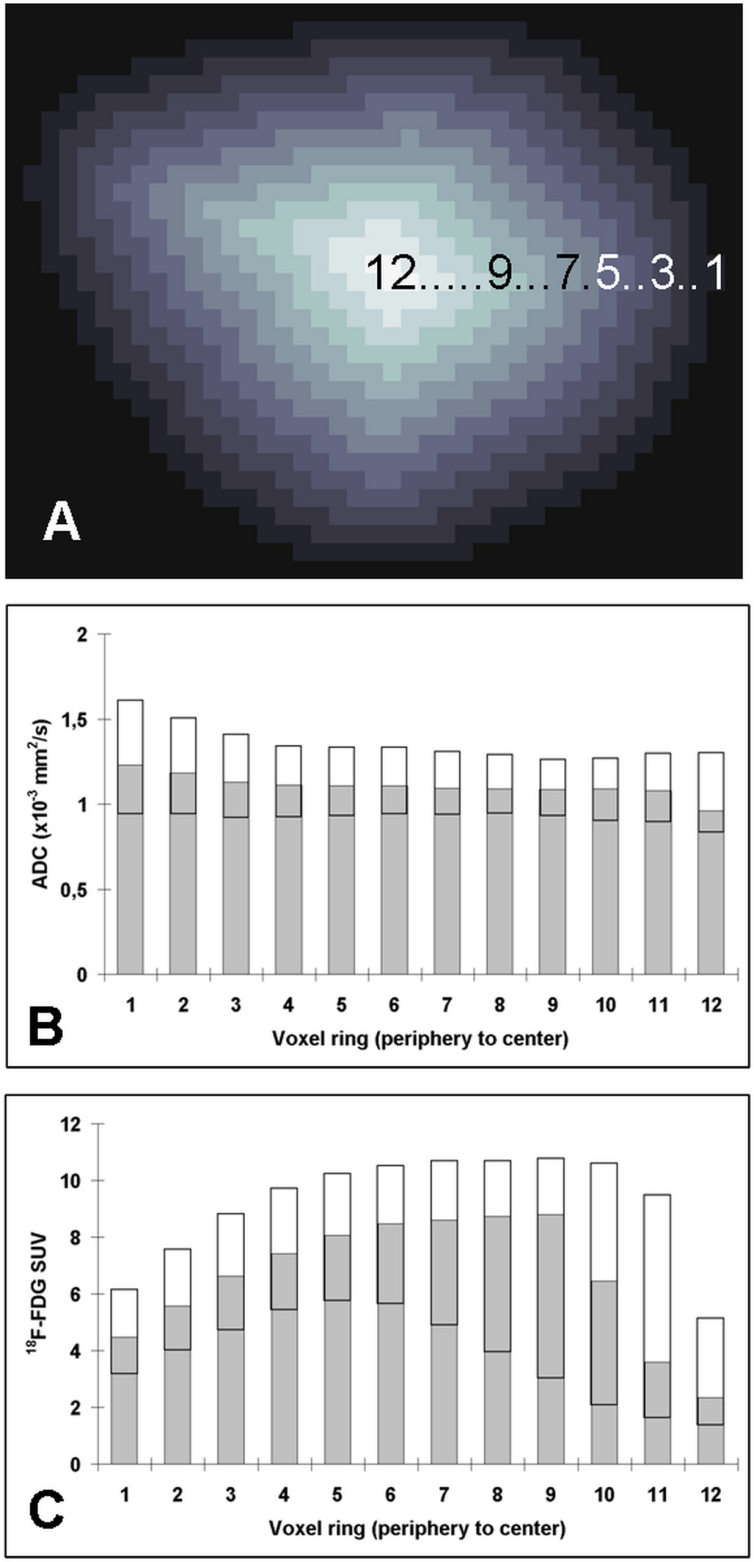
A-C. The tumors were divided into voxel rings starting from the periphery to the tumor center (A). Depending on the tumor size, up to 12 voxel rings could be defined for the largest tumors. From the entire cohort of patients, the data for each voxel ring are shown for ADC (B) and ^18^F-FDG (C) (grey bars: median; white bars: 25th to 75th percentile). Note that due to partial volume effects, there is an increase of SUV’s in the first approximately 4 voxel rings (gray-white shaded, which were omitted for further analysis), followed by a plateau. Subsequently, the SUV’s gradually decrease in the more central tumor parts. This suggests that glucose metabolism is more intense in the peripheral tumor parts and less in the tumor center. However, ADC values are more evenly distributed among the tumor.

**Fig 3:**
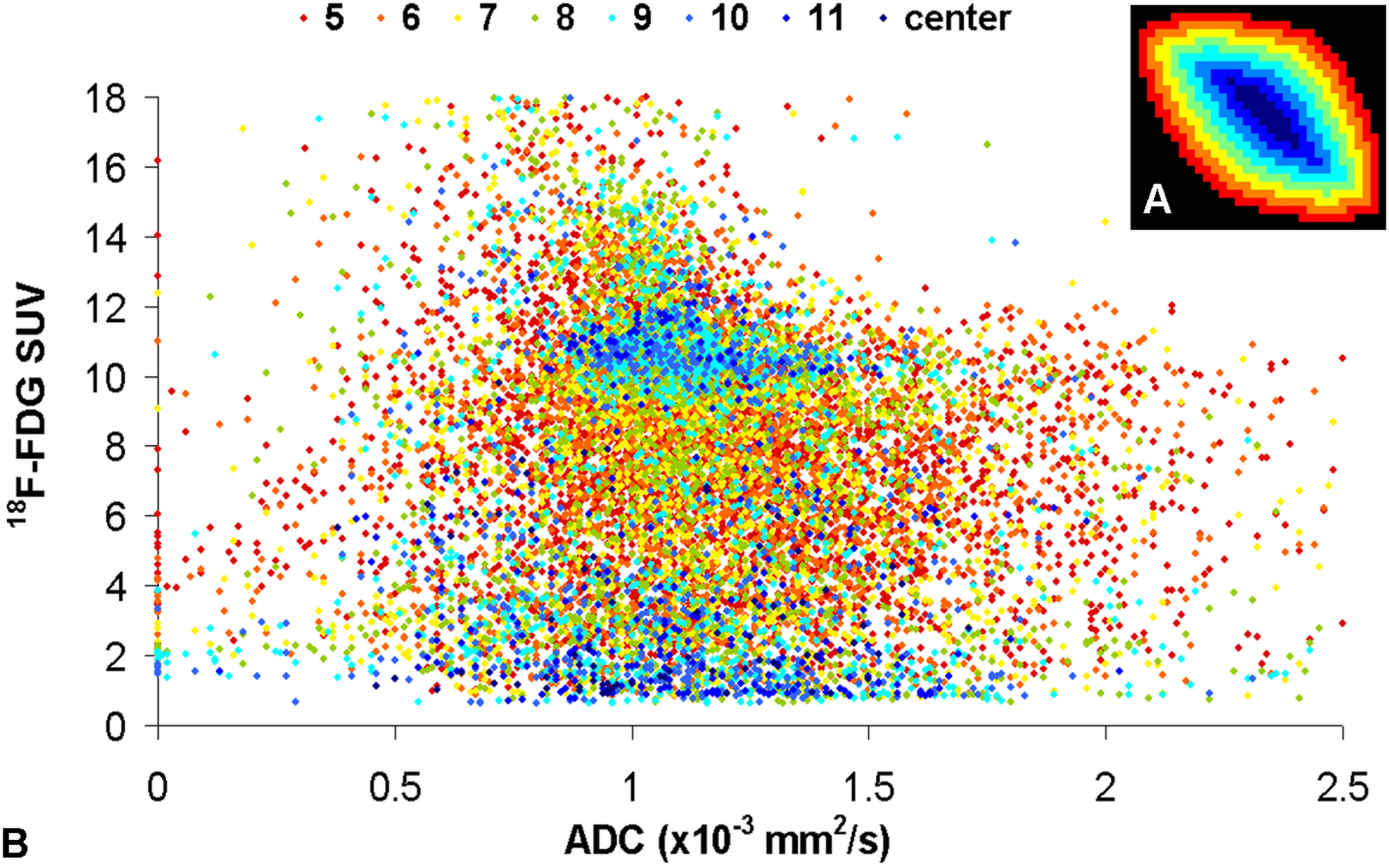
A-B. Scatter plot of the correlations of ADC and ^18^F-FDG uptake (B). The colors from (A) denote the localization of the voxels (from red: periphery to blue: center). Voxels with high uptake of ^18^F-FDG and lower ADC values can be found in all tumor parts (B). Surprisingly, there is a substantial number of voxels with low uptake of ^18^F-FDG and lower ADC values, mainly located in the central areas, maybe due to high density of cells with low tumor activity or low density of tumor cells but dense tumor stroma caused by desmoplastic reactions (B). For higher ADC values >1.5 x10-3 mm²/s the ^18^F-FDG values are widely distributed (B).

To discriminate peripheral from central tumor regions, every voxel was assigned a unique integer number, specifying the minimal distance (in voxels) from the outside of the tumor as shown in Figs 2A and 3A. The numbers were calculated by a custom made region growing algorithm, written in Matlab 7.10 (The MathWorks, Inc., Natick, MA).

### Statistical analysis

Quantitative data are presented as means and standard error of the mean or are displayed in the bar diagrams as median and 25^th^-75^th^ percentile. Distribution box-and-whisker analyses of all n = 12 different tumors revealed one outlier (far out value larger than the upper quartile plus 3 times the interquartile range) with SUV ^18^F-FDG of 17.6, which was excluded from regression analysis. For linear regression analysis, Spearman’s rank correlation coefficient r and the p-value derived from a two-tailed Student t-distribution were computed. Statistical significance was assigned for p<0.05. Computations were performed using MedCalc (MedCalc Software, Mariakerke, Belgium).

## Results

### Tumor data

A total number of 12 tumors (one per patient; n = 7 lung, n = 3 bone, n = 1 adrenal gland, and n = 1 lymph node) were evaluated with multiple sections per tumor (total n = 76, mean 6.3, range 3–10). The mean tumor size in CT imaging was 5.9 cm (range 4–10 cm). In total, 43224 voxels were assessed (S1 File) with a mean section size of 540 voxels (voxel resolution of 2.4x1.8x5 mm^3^). The mean number of voxel rings of the sections was 7.9, range 3–12. The following mean values of all voxels were determined: ADC = 1.12±0.01x10^-3^ mm²/s and SUV ^18^F-FDG = 6.9±0.2.

### Correlation analysis of the mean entire tumor data

Regarding the mean data of the whole tumor, no significant correlation was found between the ADC and molecular PET data (ADC vs. SUV ^18^F-FDG: *r* = 0.3, *P* = 0.37).

### Spatial heterogeneity analysis

The spatial distribution of the different quantitative parameters in each voxel ring from tumor periphery (voxel ring 1) to tumor center (maximum 12) is displayed in **Fig 2**. The ADC values showed a comparably homogenous distribution over the mean entire tumor area with a median range from 0.96–1.23x10^-3^ mm²/s.

Regarding radiotracer uptake, there was an increase of uptake notable in the most peripheral tumor parts, which is probably caused by partial volume effects at the border of the tumors to the surrounding tissue. Thus, the 4 most peripheral rings were excluded from voxel-by-voxel analysis to minimize systematic underestimation of peripheral SUV’s (voxel ring 1–4).

Apart from these outermost tumor parts, ^18^F-FDG tracer uptake systematically decreases towards the tumor center. In detail, the values increase initially from voxel ring 1 (median SUV = 4.5) to a maximum at voxel ring 9 (median SUV = 8.8) with a slope in the first 4 voxel rings. In the central 3 voxel rings (10–12) the values decrease to a minimum median SUV of 2.3 in the center.

### Voxel-by-voxel correlation analysis respecting spatial distribution

The first 4 voxel rings were excluded from this analysis as the SUV values might be underestimated due to partial volume effects in PET imaging. From all patients, 13723 voxels were included.

For lower ADC values <1.5x10^-3^ mm²/s, a wide distribution of the PET voxels can be noted. 9888 voxels are located in the SUV_high_ cluster (72% of all voxels), indicating vital tumor with high density of viable tumor cells. Surprisingly, 83% of the SUV_low_ cluster voxels, which are mainly located in the tumor center, also showed lower ADC values (<1.5x10^-3^ mm^2^/sec). For higher ADC values >1.5x10^-3^ mm²/s, both high and low ^18^F-FDG values can be found (Fig 3).

### Subgroup voxel-by-voxel correlation analysis for primary lung tumors only

A total number of 7 primary lung tumors only could be assessed, containing 22060 voxels. Regarding the mean data of the whole tumor, no significant correlation was found between the ADC and molecular PET data (ADC vs. SUV ^18^F-FDG: *r* = 0.25, *P* = 0.62). The following mean values of all voxels were determined: ADC = 1.21±0.02x10^-3^ mm²/s and SUV ^18^F-FDG = 7.3±0.01.

**Fig 4:**
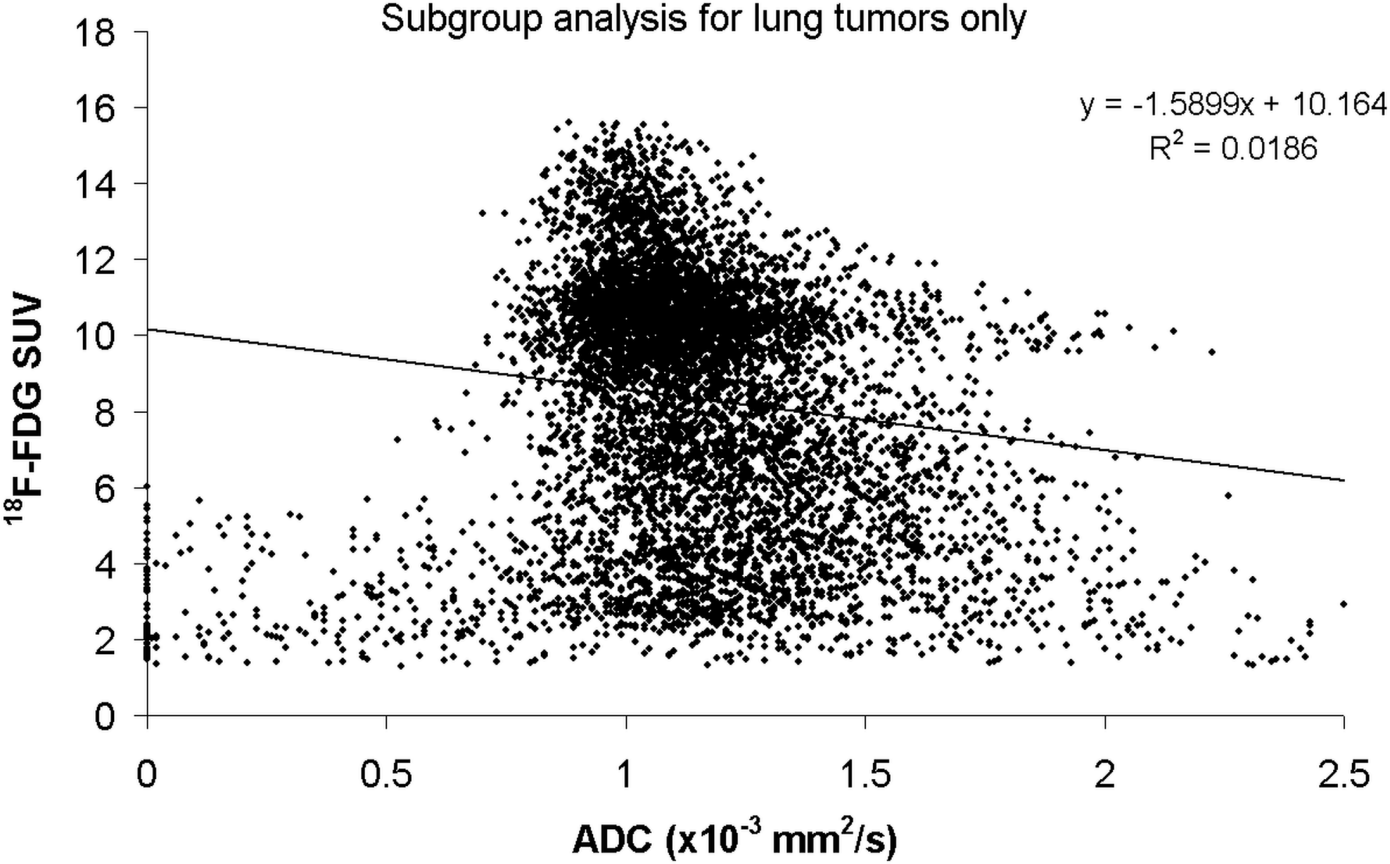
Subgroup analysis for the lung tumors only, showing a scatter plot of the voxel-by-voxel correlation of ADC and ^18^F-FDG uptake. No correlation between the ^18^F-FDG uptake and ADC can be demonstrated. The distribution of the voxel data is comparable to **Fig 3** and 75% of the voxels are located in the SUVhigh/ADClow cluster.

**Fig 5:**
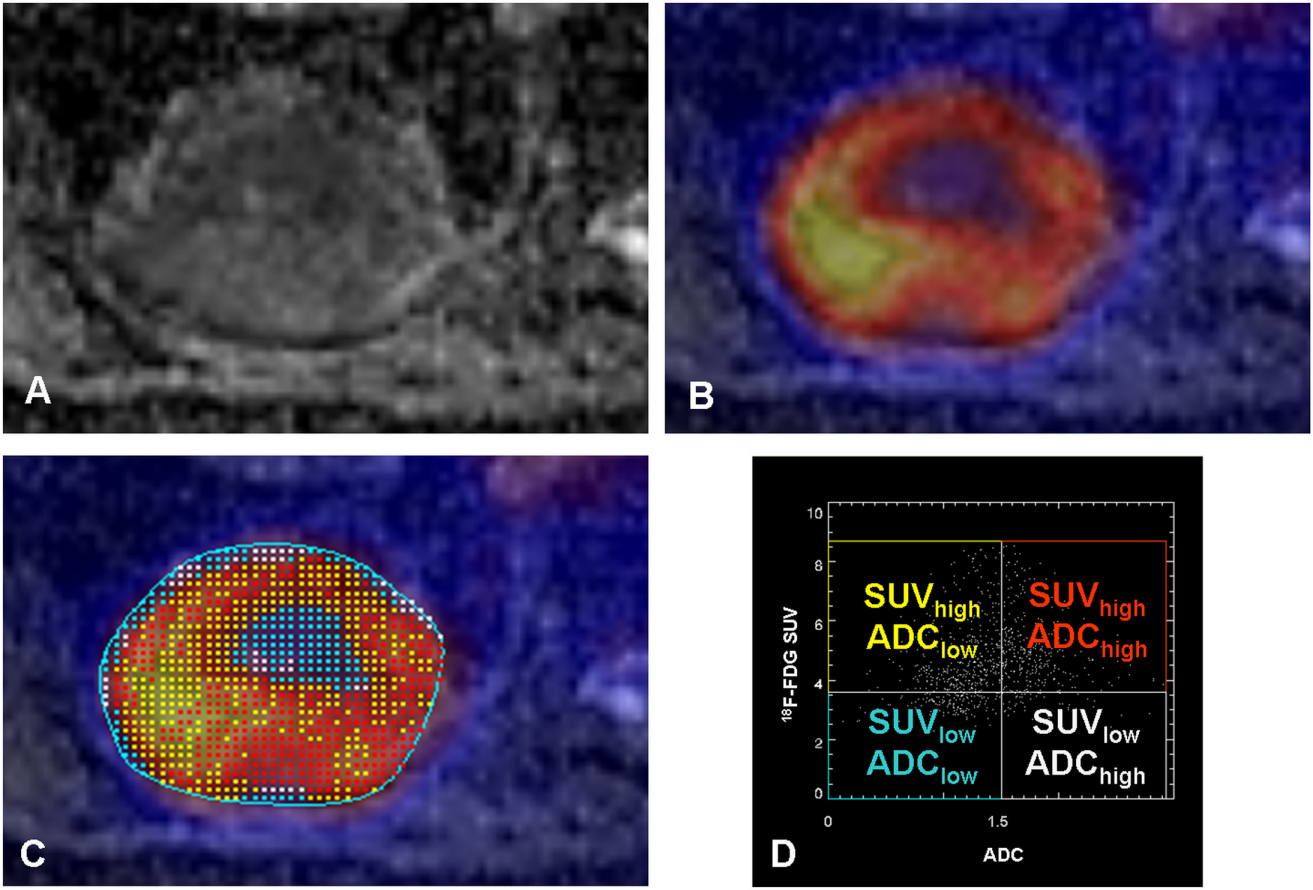
A-D. Patient with a primary NSCLC of the right lower lobe adjacent to the posterior chest wall (A: ADC map from DW-MRI). There is high ^18^F-FDG uptake in the periphery of the tumor and also the transition zone, with low uptake in the center (B image fusion of ^18^F-FDG PET and the ADC map). A ROI is placed around the tumor (C, blue circle) and the Anima M^3^P software then displays a scatter plot of the voxels within the ROI (D). Thresholds for low and high tracer uptake (y-axis) and low and high ADC values (x-axis) were defined (SUV = 3.6 for ^18^F-FDG and ADC 1.5 x10^-3^ mm²/s) and the localization of the voxels of each quadrant (yellow: SUV_high_/ADC_low_; red: SUV_high_/ADC_high_; blue: SUV_low_/ADC_low_; gray: SUV_low_/ADC_high_) can be displayed in the fused image (C). The potential biological correlate of each of the four clusters is displayed in (D).

For further analysis, the first 4 voxel rings were excluded as the SUV values might be underestimated due to partial volume effects in PET imaging. Thus, from all primary lung tumors, 8318 voxels were included. The distribution of the data points in the scatter plot is comparable to **Fig 3** for all lesions. Again, most of the voxels (75%) are located in the SUVhigh/ADClow cluster (**Fig 4**). An image example is presented in **Figs 1 and 5**.

### Cluster analysis

**Fig 6:**
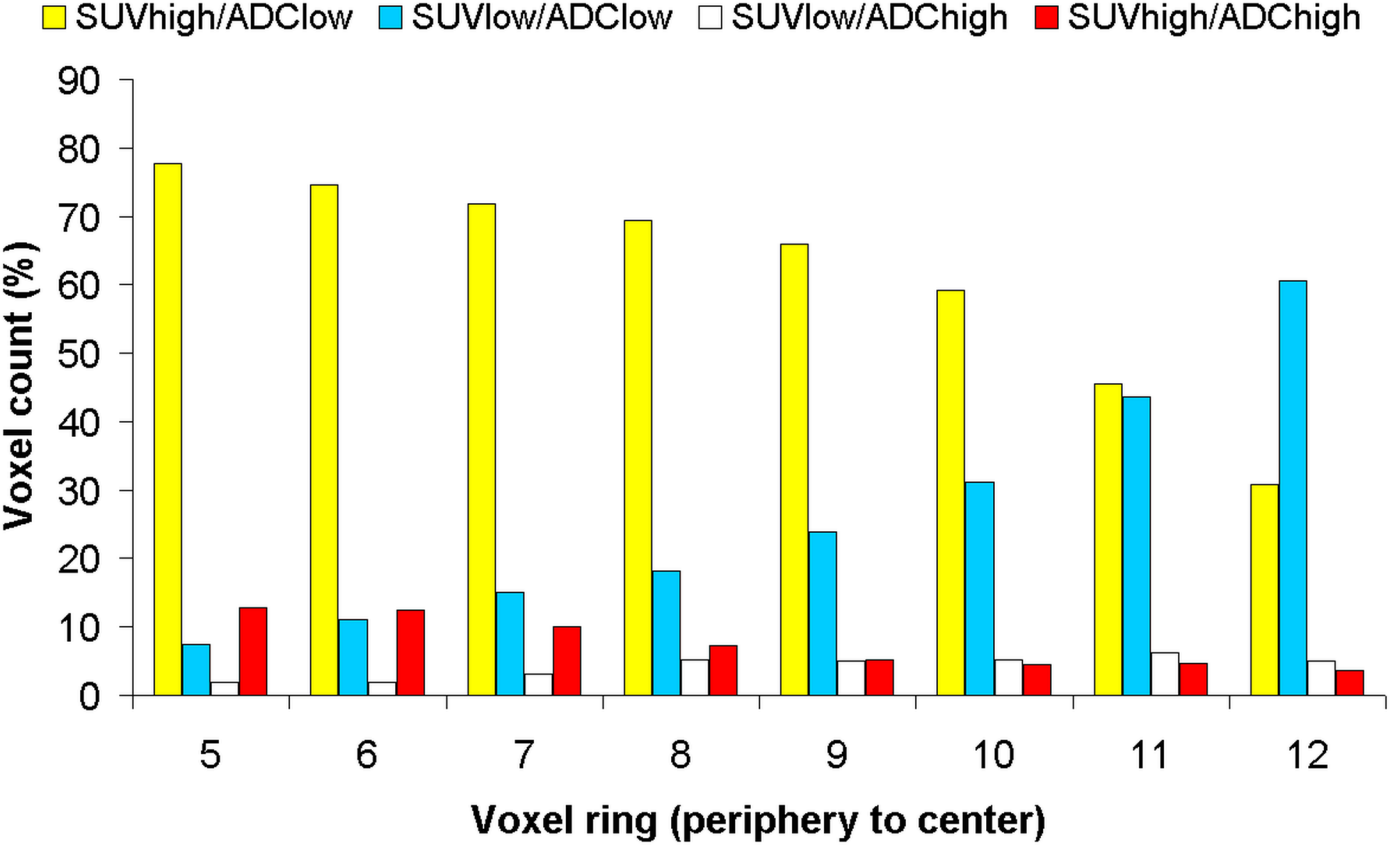
Using a threshold for low and high tracer uptake of SUV = 3.6 for ^18^F-FDG and ADC = 1.5x10^-3^ mm²/s for restricted and less restricted water diffusivity, up to four tumor cluster of different biologically activity could be defined. Here, the relative distribution (%) of the different cluster from the tumor periphery to the center is shown. The relative number of voxels with high uptake of ^18^F-FDG and lower ADC values (yellow, vital tumor) decreases to the tumor center, oppositely for voxels with low uptake of ^18^F-FDG and lower ADC values (turquoise, high density of cells with low tumor activity or low density of tumor cells but dense tumor stroma caused by desmoplastic reactions). Voxels from the clusters with less restricted water diffusivity (SUV_high_ respectively SUV_low_) can be found in all tumor parts, indicative either for hypoxia (SUV_high_, red) or necrosis (SUV_low_, white). However, the highest amount of voxels from the SUV_high_/ADC_high_ cluster can be found within the transitional zone, whereas the voxel of the SUV_low_/ADC_high_ cluster increase to central tumor parts.

Each voxel is assigned according to the thresholds to one of the four clusters (SUV_high_/ADC_low_, SUV_high_/ADC_high_, SUV_low_/ADC_low_, and SUV_low_/ADC_high_). As the total number of voxels decreases from the periphery to the center, the relative voxel counts (%) for each voxel ring are given in **Fig 6**. For SUV_high_/ADC_low_, a maximum of 78% can be found in the voxel ring 5 decreasing to the center with a minimum of 31% in voxel ring 12. Oppositely, the SUV_low_/ADC_low_ continuously increased to the center with a maximum of 60% in voxel ring 12. For SUV_low_/ADC_high_, highest values are found in central tumor parts (voxel ring 8–12). The highest amount of SUV_high_/ADC_high_ cluster voxels can be seen within the transitional zone (voxel ring 5 and 6).

## Discussion

In this report we successfully introduced a novel software platform for combined spatially resolved voxel-by-voxel analysis and visualization of quantitative MRI and PET images and tested it with DW-MRI and FDG-PET data. Our results showed that water diffusivity and glucose metabolism in NSCLC are not necessarily correlated, suggesting that the combination of DW-MRI and FDG-PET might provide complementary information on tumor biology. This novel combined parameter of tumor biology can now be analyzed and visualized by using the presented dedicated imaging software, especially for future use in hybrid PET/MR systems.

### Spatially resolved cluster analysis of PET and DW-MRI data

For analysis of the multiparametric, multimodality imaging data, we used dedicated imaging software to specifically address tumor heterogeneity on a voxel-by-voxel basis. Tissue heterogeneity is increasingly recognized as being of major importance for correctly describing tumor biology by molecular imaging [16]. When using simple ROI approaches encompassing large areas of the tumor, the signal from e.g. mostly necrotic areas and from more active and viable areas might be mixed and the resulting signal is a blend of biologically completely different areas. For the spatial analysis, the voxel growing algorithm started from the periphery to the tumor center providing voxel rings. By this approach, we summarize the data of the peripheral tumor parts regardless of the tumor size, assuming a similar biological behavior.

In our study we used the combination of ^18^F-FDG uptake and water diffusivity to define up to four areas within a given tumor with different biological characteristics: areas with high ^18^F-FDG uptake and low ADC values, with either high ^18^F-FDG and high ADC values and vice versa, and finally areas with low ^18^F-FDG uptake and high ADC values (Table 1).

**Table 1:** Relationship between the degree of water diffusivity and glucose metabolism, and their potential biological correlate.

Diffusivity of water DW-MRI	Glucose metabolism ^18^F-FDG PET	Hypothetical biological correlate
Restricted	Intense	Vital, oxygenated tumor with high density of viable tumor cells
Restricted	Low	High density of tumor cells with low activity resp. hibernating tumor cells or low density of tumor cells but dense tumor stroma caused by desmoplastic reactions
Not / less restricted	Low	Macroscopic necrosis
Not / less restricted	Moderate—intense	Lower cell density but still high glucose metabolism, maybe due to cell edema or regions of micronecrosis and potentially hypoxia

Areas with high glucose metabolism and restricted water diffusivity indicate for vital tumor with high density of viable tumor cells and an inverse correlation between the ADC from DW-MRI and the SUV from ^18^F-FDG is assumed. Accordingly, in our study, 72% of all voxels showed lower ADC values (<1.5x10^-3^ mm^2^/s) and high tracer uptake of ^18^F-FDG (SUV>3.6), but no correlation was found between the mean ADC and SUV data. The results were similar for all lesions and when analyzing only the primary tumors separately. This is in line to other results from studies with NSCLC [17], however a significant inverse correlation was found for other ADC and SUV calculations, such as ADCmin and SUVmax [17] and similar findings are reported in studies of other neoplastic lesions [18] [19] [20]. Nevertheless, the spatially resolved voxel-by-voxel cluster analysis in our study reflected the tumor heterogeneity and detected additional areas of different expressions in voxel phenotypes, which cannot be achieved by summarized data of total tumor lesions.

Furthermore, spatial distribution analysis revealed that tracer uptake of ^18^F-FDG was more pronounced in peripheral tumor parts and systematically decreased towards the tumor center demonstrating a decrease in biologic tumor activity in more central tumor parts, which is not unexpected. Usually more central tumor parts especially in larger tumors are less well perfused and might contain less active or non-viable tumor cells, explaining the decrease of ^18^F-FDG uptake. On the contrary, the ADC values were relatively homogenously distributed over the mean entire tumor area. In our study, we included chemo-naïve tumors and the distribution of the ADC values indicate not for (macro-)necrotic areas in in the more central tumor parts. Accordingly, the SUV_low_/ADC_high_ cluster, indicative for macroscopic necrosis, consisted only of a small number of voxels, still increasing towards the tumor center (see also Fig 6).

There are several potential hypothetical explanations for the presence of lower ADC values and thus restricted water diffusivity also in areas of low tumor activity and in more central tumor parts (Table 1). On the one hand, this might be caused by a relatively low number of vital tumor cells within dense tumor stroma caused by desmoplastic reactions. Another explanation might be a relatively high number of tumor cells with comparatively low glucose consumption per cell and thus lower tumor activity, e.g. due to limited perfusion and limited access to nutrients in more central tumor parts. This has to be pursued in future prospective studies with histopathological correlation.

Pronounced in the transitional zone, we found a cluster with higher ADC values >1.5x10^-3^ mm²/s and high uptake of ^18^F-FDG. This indicates that also in areas with less restricted diffusivity and thus potentially cell edema or a lower density of tumor cells, a high glucose metabolism can be found (Table 1). Again, no definite explanation can be presented, however this mismatch might be indicative for adaptation to hypoxia (“Warburg effect”). The hypoxia induced behavior of cancer cells is mediated by a heterodimeric transcription factor, hypoxia inducible factor 1α (HIF-1), leading among others to upregulated glycolysis and increased angiogenesis [21] [22]. HIF-1 increases expression of Glut-1 glucose transporters and hexokinase, which are major determinants of glucose uptake and metabolism that can be directly imaged by using ^18^F-FDG PET. This might explain that despite a potentially lower density of tumor cells, ^18^F-FDG uptake is still intense. This would also be in line with the finding that this combination was predominantly found in the transitional zone between periphery and tumor center and potentially high cell turn over, but not so much in the most central tumor parts themselves.

### Limitations

One major limitation concerning the interpretation of our data is the aspect of manual image fusion performed for data analysis in the current approach. Furthermore, due to different patient positions in MRI and PET, exact congruence of the different tumor areas analyzed cannot be guaranteed, potentially limiting the interpretation of our data. Furthermore, no proper respiratory motion correction was used for PET imaging. Also, the small number of patients included to our study and the assessment of primary tumors as well as metastases restricts the power of our results. However, many of the limitations might be overcome in the future by the recent introduction of hybrid whole-body PET/MR scanners [8].

### Conclusions and Perspectives

Multimodality image fusion and multiparametric voxel-by-voxel analysis of tumors is feasible with the dedicated imaging software used in our study. Our approach to handle such multiparametric datasets included a spatially resolved voxel-by-voxel and cluster analysis. Such multiparametric indices of tumor biology might be of clinical relevance with respect to a better evaluation of tumor invasiveness, metastatic potential and patient prognosis and might be of interest for targeting biopsies or radiation therapy planning. Many of the limitations of retrospective image fusion used in this study might be overcome by combined PET/MR scanners, which are increasingly used preclinically and clinically [23].

## Supporting Information

S1 File Dataset of voxels. (TXT)
